# Evolutionary Processes in Quantum Decision Theory

**DOI:** 10.3390/e22060681

**Published:** 2020-06-18

**Authors:** Vyacheslav I. Yukalov

**Affiliations:** 1 Bogolubov Laboratory of Theoretical Physics, Joint Institute for Nuclear Research, 141980 Dubna, Russia; yukalov@theor.jinr.ru; Tel.: +7-496-216-3947; 2 Department of Management, Technology and Economics, ETH Zürich, Swiss Federal Institute of Technology, CH-8032 Zürich, Switzerland

**Keywords:** quantum decision theory, operationally testable choice, dual decision process, quantum-classical correspondence, quantum intelligence network

## Abstract

The review presents the basics of quantum decision theory, with an emphasis on temporary processes in decision making. The aim is to explain the principal points of the theory. How an operationally-testable, rational choice between alternatives differs from a choice decorated by irrational feelings is elucidated. Quantum-classical correspondence is emphasized. A model of quantum intelligence network is described. Dynamic inconsistencies are shown to be resolved in the frame of the quantum decision theory.

## 1. Introduction

In recent years, there has been great interest in the possibility of formulating decision theory in the language of quantum mechanics. Numerous references on this topic can be found in the books [1,2,3,4] and review articles [5,6,7,8]. This interest is caused by the inability of classical decision theory [9] to comply with the behavior of real decision makers, which requires the development of other approaches. Resorting to the techniques of quantum theory gives hope for a better representation of behavioral decision making. There are several variants of using quantum mechanics for interpreting conscious effects. The aim of the present review is not the description of the existing variants, which would need too much space and can be found in the cited literature [1,2,3,4,5,6,7,8], but a survey of the approach suggested by the author and his coworkers. This approach was coined [10] *quantum decision theory* (QDT).

In the present review, we limit ourselves by considering quantum decision theory, but we do not touch other trends in the ramified applications of quantum techniques, such as quantum approaches in physics, chemistry, biology, economics and finance, quantum information processing, quantum computing, and quantum games. It is evident that all those fields cannot be reasonably described in a single review.

Although the theory of quantum games shares similarities with decision theory, there exists an important difference between the standard treatment of quantum games [11,12,13,14,15] and the main idea of quantum decision theory presented in the review. In the theory of quantum games, one usually assumes that players are quantum devices of a kind following quantum rules [16,17]. However, in the approach of quantum decision theory [10], decision makers do not have to be quantum devices; moreover, they can be real human beings. The mathematics of QDT is analogous to the mathematics in the theory of quantum measurements, where an observer is a classical human being, while the observed processes are characterized by quantum laws. In QDT, quantum theory is a technical language allowing for the description of decision processes. Quantum techniques turn out to be a very convenient tool for characterizing realistic human decision processes incorporating rational–irrational duality, because quantum techniques are designed for taking into account the dual nature of quantum world, such as the particle–wave duality. The mathematical generalization of decision-making processes by including into them the rational–irrational duality is one of the main achievements of QDT. Summarizing, the specific features of QDT, distinguishing it from many variants of decision theory employing quantum techniques, are as follows.

(i) The mathematics of QDT is analogous to that used for characterizing quantum measurements, so that there is a direct correspondence between QDT and the theory of quantum measurements. (ii) The approach is general, not only in the description of both decision theory and quantum measurements, but also in its mathematical formulation, allowing for the interpretation of various decision events or quantum measurements. (iii) The theory shows the difference between the decision making with respect to operationally testable events and the choice under uncertainty caused by irrational subconscious feelings. (iv) Single decisions and successive decisions are described on equal footing. (v) Temporal evolution of decision processes is formulated. (vi) Quantum-classical correspondence is preserved explaining the relation between quantum and classical probabilities. (vii) No paradoxes of classical decision theory arise. (viii) Different dynamic decision inconsistencies find natural explanation in the frame of QDT. (ix) The theory can be applied to single decision makers and also to decision-maker societies forming quantum intelligence networks.

Of course, not all above-mentioned aspects will and can be considered in detail in the present review. To describe all of them would require a huge book. We shall concentrate on the principal points the theory is based on and will emphasize temporal effects that have not been sufficiently discussed in the previous publications. In order to make clear for the reader the basic ideas of the QDT, it is useful to present the foundations in several steps. First, we need to define the process of decision making in the case of operationally testable events. This introduces the method of calculating quantum probabilities in the case of projection valued measures. Then it is straightforward to generalize the considerations to dual processes, when a decision is made by taking into account rational and irrational sides, which involves the use of positive operator-valued measures. Next, the approach to describing a society of decision makers, forming a quantum intelligence network, is presented. Finally, several dynamic decision inconsistencies are considered as an illustration of how they can be naturally resolved in the frame of QDT.

## 2. Operationally Testable Events

The mathematics of QDT is similar to that employed for treating quantum measurements. Therefore, each formula can be interpreted from two angles—from the point of view of quantum measurements or decision theory [10,18,19,20]. Below, as a rule, we follow the interpretation related to decision theory, only occasionally mentioning quantum measurements. Some parts of the scheme below are familiar to quantum mechanics, but here we suggest the interpretation in the language of decision theory, which is needed for introducing the basic notions of QDT. These basic notions are introduced step by step in order to better demonstrate the logic of the QDT. We will not plunge into the details of numerous applications that can be found in the published literature. The main aim of this review is the explanation of the principal points of the approach, with the emphasis on the evolutionary side of decision processes.

The typical illustration of decision making is based on the choice between given alternatives, say forming a set $\left\{A_{n}\right\}$, enumerated by the index $n=1,2,\ldots ,N_{A}$. The alternatives correspond to events that can be operationally tested. Each alternative is represented by a vector $|A_{n}\rangle$, where the bra-ket notation [21] is used. The vectors of alternatives pertain to a Hilbert *space of alternatives*

(1) $$H_{A}=\mathrm{span}\;\{\;|\;n\;\rangle \;\},$$

being a closed linear envelope over an orthonormal basis. Decisions are made by a subject whose mind is represented by a Hilbert space

(2) $$H_{S}=\mathrm{span}\;\{\;|\;\alpha \;\rangle \;\}$$

that can be called the *subject space of mind*, or just the *subject space*. Thus, the total space where decisions are made is the *decision space*

(3) $$H=H_{A}⨂H_{S}.$$

This is a closed linear envelope over an orthonormal basis,

H=span { | nα 〉≡| n 〉⊗| α 〉 }.

The alternatives from the given set, represented by vectors $|A_{n}\rangle$, are assumed to be orthonormal to each other,

$$\langle \;A_{m}\;|\;A_{n}\;\rangle =\delta_{mn},$$

which means that the alternatives are mutually exclusive. The alternative vectors are not required to form a basis. For each alternative, it is possible to show a corresponding alternative operator

(4) $$\hat{P}\left(A_{n}\right)=|\;A_{n}\;\rangle \langle \;A_{n}\;|.$$

These operators enjoy the properties of projectors

$$\hat{P}\left(A_{m}\right)\hat{P}\left(A_{n}\right)=\delta_{mn}\hat{P}\left(A_{n}\right),\;\;\sum_{n}\hat{P}\left(A_{n}\right)=1,$$

whose family forms a projection-valued measure.

The state of a decision maker is represented by a statistical operator that is a semi-positive trace-one operator,

(5) $${\mathrm{Tr}}_{H}\;\hat{\rho }\left(t\right)=1,$$

depending on time *t* and acting on the space H. In decision making, $\hat{\rho }$ is termed the decision-maker state or just the decision state. The pair {H,ρ(t)} is a *decision ensemble*. The probability of observing an event $A_{n}$ is

(6) $$p\left(A_{n},t\right)={\mathrm{Tr}}_{H}\;\hat{\rho }\left(t\right)\hat{P}\left(A_{n}\right),$$

which is uniquely defined for a Hilbert space of dimensionality larger than two [22]. Since $\hat{P}\left(A_{n}\right)$ acts on the space $H_{A}$, probability (6) can be rewritten as

(7) $$p\left(A_{n},t\right)={\mathrm{Tr}}_{A}\;{\hat{\rho }}_{A}\left(t\right)\hat{P}\left(A_{n}\right),$$

where the trace is over the space $H_{A}$, and

(8) $${\hat{\rho }}_{A}\left(t\right)={\mathrm{Tr}}_{S}\;\hat{\rho }\left(t\right)=\sum_{\alpha }\langle \;\alpha \;|\;\hat{\rho }\left(t\right)\;|\;\alpha \;\rangle ,$$

with the trace over the space $H_{S}$. Note that probability (7) can also be written as

$$p\left(A_{n},t\right)={\mathrm{Tr}}_{A}\;\hat{P}\left(A_{n}\right){\hat{\rho }}_{A}\left(t\right)\hat{P}\left(A_{n}\right).$$

Expression (6), or (7), is the predicted probability of observing an event $A_{n}$, or in decision theory, this is the predicted probability of choosing an alternative $A_{n}$. When comparing the predicted theoretical probabilities with empirical probabilities, the former are interpreted in the frequentist framework [23,24]. Thus, $p(A_{n},t)$ is the fraction of equivalent decision makers preferring the alternative $A_{n}$ at time *t*. This can be reformulated in the different way: $p(A_{n},t)$ is the frequency of choosing the alternative $A_{n}$ by a decision maker, if the choice is repeated many times under the same conditions until the time *t*.

We say that an alternative $A_{i}$ is stochastically preferred (or simply preferred) to an alternative $A_{j}$ if and only if $p\left(A_{i}\right)>p\left(A_{j}\right)$, and the alternatives $A_{i}$ and $A_{j}$ are stochastically indifferent if and only if $p\left(A_{i}\right)=p\left(A_{j}\right)$.

## 3. Single Decision Making

Usually, one considers decision making as a process instantaneous in time, which is certainly an overly rough modeling. Here we stress the importance of dealing with realistic decision making developing in time. The evolutionary picture saves us from different inconsistencies arising in the toy model of instantaneous decisions [18,25].

Suppose at the initial time t=0 we plan to make a choice between the alternatives from the given set. This implies a stage of preparation, when the decision maker is characterized by a state $\hat{\rho }\left(0\right)$. The evolution of the decision-maker state in time can be represented as

(9) $$\hat{\rho }\left(t\right)=\hat{U}\left(t\right)\;\hat{\rho }\left(0\right)\;{\hat{U}}^{+}\left(t\right),$$

involving the evolution operator $\hat{U}\left(t\right)$. To keep the state normalization intact, the evolution operator has to be unitary,

$${\hat{U}}^{+}\left(t\right)\hat{U}\left(t\right)=1.$$

By employing a self-adjoint evolution generator $\hat{H}\left(t\right)$, the time transformation of the evolution operator can be written as the Lie differential equation

(10) $$\frac{d}{dt}\;\hat{U}\left(t\right)=\hat{H}\left(t\right)\hat{U}\left(t\right),$$

with the initial condition

(11) $$\hat{U}\left(0\right)=\hat{1}.$$

A very important clause is the requirement that the considered decision makers be well defined individuals, who do not drastically vary in time; otherwise, there would be no meaning in predicting their decisions. In other words, the mental features of decision makers at one moment of time are to be similar to those at a different moment of time. Briefly speaking, we can say that the decision makers should have the property of self-similarity. This imposes a restriction on the form of the evolution generator. This restriction can be written as the commutator

(12) $$\left[\hat{H}\left(t\right),\;\int_{0}^{t}\hat{H}\left(t^{\prime }\right)dt^{\prime }\right]=0.$$

Recall that in quantum theory the operator commuting with the evolution generator is called the integral of motion. In our case, condition (12) does not mean that the evolution generator does not depend on time, but it tells us that the properties of this operator are in some sense invariant with time, preserving the similarity of the decision makers at different moments of time. Thus, in decision theory, condition (12) can be understood as the invariance of the properties of decision makers; that is, of the *decision-maker self-similarity*. In the theory of operator differential equations, commutator (12) is termed the Lappo-Danilevsky condition [26]. Under this provision, the evolution operator, satisfying the evolution Equation (10), with the initial condition (11), reads as

(13) $$U\left(t\right)=\exp\left\{-i\int_{0}^{t}\hat{H}\left(t^{\prime }\right)\;dt^{\prime }\right\}.$$

This form of the evolution operator satisfies the group properties that can be represented as the property of functional self-similarity [27].

Till now, we have not specified the choice of a basis for the decision space H. It is convenient to accept as the basis that one composed of the eigenfunctions of the evolution generator, such that

(14) $$\hat{H}\left(t\right)\;|\;n\alpha \;\rangle =E_{n\alpha }\left(t\right)\;|\;n\alpha \;\rangle .$$

Generally, the eigenfunctions here can depend on time. However, there are two cases when this dependence is either negligible or absent at all. One possibility is when the eigenfunctions vary with time much slower than the eigenvalues $E_{n\alpha }\left(t\right)$. Then there exists a time horizon till which the time variation of the eigenfunction can be neglected, which is regulated by a kind of adiabatic conditions [28]. The other case is the already assumed Lappo-Danilevsky condition (12) implying the decision-maker self-similarity. It is easy to show that the Lappo-Danilevsky condition (12) is equivalent to the validity of eigenproblem (14) with time-independent eigenfunctions. Exactly the same situation happens in quantum theory in the case of nondestructive or nondemolition measurements [29,30,31,32,33,34,35].

This basis is complete, since the evolution generator is self-adjoint. Then

(15) $$\hat{U}\left(t\right)\;|\;n\alpha \;\rangle =U_{n\alpha }\left(t\right)\;|\;n\alpha \;\rangle ,$$

where

(16) $$U_{n\alpha }\left(t\right)=\exp\left\{-i\int_{0}^{t}E_{n\alpha }\left(t^{\prime }\right)\;dt^{\prime }\right\}.$$

In this way, we find the expression for the time-dependent predicted probability of choosing the alternative $A_{n}$ at time *t*,

(17) $$p\left(A_{n},t\right)=\sum_{n_{1}n_{2}}\;\sum_{\alpha }U_{n_{1}\alpha }\left(t\right)\langle \;\alpha n_{1}\;|\;\hat{\rho }\left(0\right)\;|\;n_{2}\alpha \;\rangle \;U_{n_{2}\alpha }^{*}\left(t\right)\langle \;n_{2}\;|\;\hat{P}\left(A_{n}\right)\;|\;n_{1}\;\rangle .$$

Again, to compare this expression with experimental observations, it is reasonable to interpret it as a frequentist probability [23,24], although other interpretations are admissible [36].

The evolution generator is defined on the decision space H and characterizes the velocity of the change of the decision-maker state caused by the influence of the set of alternatives. The impact of the evolution generator on the decision-maker state is assumed to be finite, such that, if the decision process starts at time $t_{A}$ and ends at time $t_{A}+\tau_{A}$, being taken in the time interval $\left[t_{A},t_{A}+\tau_{A}\right]$, then

(18) $$\left|\int_{t_{A}}^{t_{A}+\tau_{A}}E_{n\alpha }\left(t\right)\;dt\;\right|<\infty .$$

This impact can be asymptotically large, remaining finite. The speed of the decision process can be quantified by a rate parameter *g*, which the eigenvalue $E_{n\alpha }\left(t\right)=E_{n\alpha }\left(t,g\right)$ depends on in such a way that in the limit of a slow process

(19) $$E_{n\alpha }\left(t,g\right)\to 0\;\;\left(g\to 0\right),$$

while under a fast process

(20) $$E_{n\alpha }\left(t,g\right)\to \infty \;\;\left(g\to \infty \right).$$

In the case of a slow process,

$$U_{n\alpha }\left(t\right)≃1\;\;\left(g\to 0\right).$$

This yields the probability

(21) $$p\left(A_{n},t\right)≃{\mathrm{Tr}}_{A}\;{\hat{\rho }}_{A}\left(0\right)\hat{P}\left(A_{n}\right)\;\;\left(g\to 0\right),$$

which implies that the decision state practically does not change:

(22) $${\hat{\rho }}_{A}\left(t\right)≃{\hat{\rho }}_{A}\left(0\right)\;\;\left(g\to 0\right).$$

When the process is fast, in the sum (17) one has

$$U_{m\alpha }\left(t\right)\;U_{n\alpha }^{*}\left(t\right)≃\delta_{mn}\;\;\left(g\to \infty \right).$$

This leads to the probability

(23) $$p\left(A_{n},t\right)≃{\mathrm{Tr}}_{A}\;{\hat{\rho }}_{A}\left(t\right)\hat{P}\left(A_{n}\right)\;\;\left(g\to \infty \right),$$

with the state

(24) $${\hat{\rho }}_{A}\left(t\right)≃\sum_{m}{\hat{P}}_{m}\;{\hat{\rho }}_{A}\left(0\right)\;{\hat{P}}_{m}\;\;\left(g\to \infty \right),$$

where

$${\hat{P}}_{m}\equiv |\;m\;\rangle \langle \;m\;|.$$

In the intermediate cases of the process rate, one has to resort to the general probability (17).

## 4. Changing Initial Conditions

Assume that a subject made a decision during the time interval $\left[t_{A},t_{n}\equiv t_{A}+\tau_{A}\right]$ choosing the alternative $A_{n}$. Then what would be the following evolution of the probability $p(A_{n},t)$? Strictly speaking, there is nothing special in the fact of one subject making a decision at any moment of time. According to the frequentist understanding, the probability gives a distribution over an ensemble of decision makers or over many repeated decisions. This means that the probability yields a fraction of decision makers (or the fraction of decisions) preferring this or that alternative. In the following moments of time, the probability will continue to be defined by Equation (17).

However, one can put the question in a different manner: Suppose we are interested in the decision making of just a single subject who certainly chose the alternative $A_{n}$, so that at the moment $t_{n}$ the probability became one, instead of that prescribed by Equation (17). Then what would be the following evolution of the probability? Again, there is nothing extraordinary in that case. Before making a decision, the predicted probability was described by Equation (17). After making the decision, if we insist that a posteriori probability became one, this means that we have to replace $p(A_{n},t_{n})$ by one, treating the latter as a new initial condition. Thus, the replacement

(25) $$p(A_{n},t_{n})⟼1$$

means nothing but the change of the initial condition for the equation describing the evolution of the decision state. The related replacement

(26) $${\hat{\rho }}_{A}\left(t_{n}\right)\;⟼\;{\hat{\rho }}_{L}\left(t_{n}\right)$$

assumes that starting from the moment of time $t_{n}$, we treat as the new initial condition the state defined by the equality

(27) $${\mathrm{Tr}}_{A}\;{\hat{\rho }}_{L}\left(t_{n}\right)\hat{P}\left(A_{n}\right)=1.$$

The simplest solution for the latter equation is the Lüders state [37]

(28) $${\hat{\rho }}_{L}\left(t_{n}\right)=\frac{\hat{P}\left(A_{n}\right){\hat{\rho }}_{A}\left(t_{n}\right)\hat{P}\left(A_{n}\right)}{{\mathrm{Tr}}_{A}\;{\hat{\rho }}_{A}\left(t_{n}\right)\hat{P}\left(A_{n}\right)}.$$

Note that here ${\hat{\rho }}_{L}\left(t_{n}\right)$ is the a posteriori post-decision state, while ${\hat{\rho }}_{A}\left(t_{n}\right)$ is the a priori anticipated state; that is,

$${\hat{\rho }}_{L}\left(t_{n}\right)\equiv {\hat{\rho }}_{L}\left(t_{n}+0\right),\;\;{\hat{\rho }}_{A}\left(t_{n}\right)\equiv {\hat{\rho }}_{A}\left(t_{n}-0\right).$$

The replacement (26) often is labeled “collapse”. For wave functions, this replacement is equivalent to the sudden change from a state |ψ〉 to the state $|A_{n}\rangle$. It is really easy to check that the wave function “collapse” implies the replacement

$${\hat{\rho }}_{A}\left(t_{n}\right)=|\;\psi \;\rangle \langle \;\psi \;|\;⟼\;{\hat{\rho }}_{L}\left(t_{n}\right)=|\;A_{n}\;\rangle \langle \;A_{n}\;|.$$

Under the name “collapse”, one means a sudden jump of the state, which can be dramatic, provided the wave function or decision state describes real matter. However, a decision state and a wave function are nothing but the probabilistic characteristics that can be used for calculating and predicting a priori quantum probabilities satisfying evolution equations. Fixing a decision state at some moment of time simply means fixing new initial conditions for the state evolution. Thus, the Lüders state is just the new initial condition for the moment of time $t_{n}$,

(29) $${\hat{\rho }}_{L}\left(t_{n}\right)={\mathrm{Tr}}_{S}\;\hat{\rho }\left(t_{n},t_{n}\right),$$

after which again the predicted probabilities have to be found from the state evolution

(30) $$\hat{\rho }\left(t,t_{n}\right)=\hat{U}\left(t,t_{n}\right)\;\hat{\rho }\left(t_{n},t_{n}\right)\;{\hat{U}}^{+}\left(t,t_{n}\right),$$

with the evolution operator

(31) $$\hat{U}\left(t,t_{n}\right)=\exp\left\{-i\int_{t_{n}}^{t}\hat{H}\left(t^{\prime }\right)\;dt^{\prime }\right\}.$$

This defines the decision state

(32) $${\hat{\rho }}_{A}\left(t,t_{n}\right)={\mathrm{Tr}}_{S}\;\hat{\rho }\left(t,t_{n}\right)\;\;\left(t>t_{n}\right)$$

after the time $t_{n}$. Respectively, this decision state gives the probability of choosing an alternative $A_{m}$ after the time $t_{n}$,

(33) $$p\left(A_{m},t\right)={\mathrm{Tr}}_{A}\;{\hat{\rho }}_{A}\left(t,t_{n}\right)\;\hat{P}\left(A_{m}\right)\;\;\left(t>t_{n}\right).$$

Comparing Equations (29) and (32) yields the initial condition

(34) $${\hat{\rho }}_{A}\left(t_{n},t_{n}\right)={\hat{\rho }}_{L}\left(t_{n}\right).$$

Following the same steps as in the previous section, we get the time evolution of the probability for $t>t_{n}$,

(35) $$\begin{array}{c} p\left(A_{m},t\right)=\sum_{n_{1}n_{2}}\;\sum_{\alpha }U_{n_{1}\alpha }\left(t,t_{n}\right)\langle \;\alpha n_{1}\;|\;\hat{\rho }\left(t_{n},t_{n}\right)\;|\;n_{2}\alpha \;\rangle \times \\ \times U_{n_{2}\alpha }^{*}\left(t,t_{n}\right)\langle \;n_{2}\;|\;\hat{P}\left(A_{m}\right)\;|\;n_{1}\;\rangle \;\;\left(t>t_{n}\right), \end{array}$$

where

$$U_{n\alpha }\left(t,t_{n}\right)=\exp\left\{-i\int_{t_{n}}^{t}E_{n\alpha }\left(t^{\prime }\right)\;dt^{\prime }\right\}.$$

In the limit of a slow process, this results in

(36) $${\hat{\rho }}_{A}\left(t,t_{n}\right)≃{\hat{\rho }}_{L}\left(t_{n}\right)\;\;\left(g\to 0\right).$$

And under a fast process, we have

(37) $${\hat{\rho }}_{A}\left(t,t_{n}\right)≃\sum_{m}{\hat{P}}_{m}\;{\hat{\rho }}_{L}\left(t_{n}\right)\;{\hat{P}}_{m}\;\;\left(g\to \infty \right).$$

Let us consider the situation, when a subject, after fixing the initial condition (29), where the alternative $A_{n}$ was chosen, is interested in finding the probability of deciding on the alternative $A_{m}$. In the slow-process limit, we get

(38) $$p\left(A_{m},t\right)={\mathrm{Tr}}_{A}\;{\hat{\rho }}_{L}\left(t_{n}\right)\;\hat{P}\left(A_{m}\right)\equiv p_{L}\left(A_{m},t_{n}\right).$$

Introducing the Wigner [38] probability

(39) $$p_{W}\left(A_{m},t_{n}\right)={\mathrm{Tr}}_{A}\;\hat{P}\left(A_{n}\right)\;{\hat{\rho }}_{A}\left(t_{n}\right)\;\hat{P}\left(A_{n}\right)\;\hat{P}\left(A_{m}\right)$$

yields the relation

(40) $$p_{W}\left(A_{m},t_{n}\right)=p_{L}\left(A_{m},t_{n}\right)\;p\left(A_{n},t_{n}\right),$$

where

$$p\left(A_{n},t_{n}\right)={\mathrm{Tr}}_{A}\;{\hat{\rho }}_{A}\left(t_{n}\right)\;\hat{P}\left(A_{n}\right).$$

This equation reminds us the relation between the classical joint and conditional probabilities. Therefore, sometimes one is tempted to interpret the Wigner probability, $p_{W}\left(A_{m},t_{n}\right)$ as a joint probability of two events, $A_{n}$ and $A_{m}$, and the Lüders probability $p_{L}\left(A_{m},t_{n}\right)$ as a conditional probability of the event $A_{m}$ under the event $A_{n}$ that previously happened. This interpretation, however, cannot pretend to provide a generalization of classical probabilities to quantum theory. This, first of all, because the derived relation is a very particular case of extremely slow processes, when g→0, but not the general expression. Second, this relation is not valid for all times, but it only connects the terms at time $t_{n}$. What is more important is that this relation contains the terms taken from different sides of $t_{n}$; that is, connecting the predicted a priori probability with the post-decision a posteriori probability,

$$p_{L}\left(A_{m},t_{n}+0\right)=\frac{p_{W}\left(A_{m},t_{n}-0\right)}{p(A_{n},t_{n}-0)}.$$

But the meaningful definition of a probability should be valid for any time $t>t_{n}$. Third, the generalization of a classical expression assumes to contain the classical one as a particular case. But it is easy to check that the Lüders probability is

$$p_{L}\left(A_{m},t_{n}\right)={\left|\;\langle \;A_{m}\;|\;A_{n}\;\rangle \;\right|}^{2},$$

which is symmetric with respect to the interchange between $A_{m}$ and $A_{n}$. For commuting events, corresponding to the classical case, the Lüders probability becomes trivial $\delta_{mn}$. Contrary to this, the classical conditional probability is neither symmetric nor trivial, which is confirmed by numerous empirical observations [39,40]. In the best case, the Lüders probability is just a transition probability [18,25].

Sometimes one tries to save the interpretation of the Lüders probability as a conditional probability by resorting to the assumption of degenerate quantum states. This, nevertheless, does not save the situation because of several reasons: The origin of degeneracy is never defined, which makes the assumption groundless. The degeneracy is not important, since it always can be lifted by infinitesimally small variations of the problem [18,25]. Finally, the degeneracy does not make classical expressions a particular case of quantum formulae; i.e., there is no quantum-classical correspondence [18,25,41].

As we see, the consideration of realistic decision processes developing across time helps us to avoid misrepresentation of the obtained expressions. The use of formal relations, neglecting evolutionary processes, can lead to meaningless results like negative and complex probabilities [18,25,42].

## 5. Successive Decision Making

When we consider the alternatives from the same set, say $\left\{A_{n}\right\}$, the probability of any $A_{n}$ is described by the approach elucidated in the previous sections, which leads to the set of the probabilities $\left\{p\left(A_{n},t\right):n=1,2,\ldots ,N_{A}\right\}$. A rather different problem is the definition of successive decisions with respect to alternatives from different sets. Below we consider this problem by employing some of the methods from the theory of quantum measurements [21,42,43,44], essentially modifying them, however, in order to adjust to quantum decision theory [18,25].

Suppose there are two sets of alternatives, $\left\{A_{n}:n=1,2,\ldots ,N_{A}\right\}$ and $\left\{B_{k}:k=1,2,\ldots ,N_{B}\right\}$. At the time interval $\left[t_{A},t_{A}+\tau_{A}\right]$, a subject makes a decision with respect to the alternatives $A_{n}$. Then, in the time interval $\left[t_{B}+\tau_{B}\right]$, where $t_{B}>t_{A}+\tau_{A}$, the subject decides about $B_{k}$.

According to the general approach of defining quantum joint probabilities [18,25], each set of alternatives is represented by the related set of alternative vectors and alternative projection operators,

(41) $$\begin{array}{c} A_{n}\;⟼\;|\;A_{n}\;\rangle \;⟼\;\hat{P}\left(A_{n}\right)\equiv |\;A_{n}\;\rangle \langle \;A_{n}\;|,\\ B_{k}\;⟼\;|\;B_{k}\;\rangle \;⟼\;\hat{P}\left(B_{k}\right)\equiv |\;B_{k}\;\rangle \langle \;B_{k}\;|. \end{array}$$

Respectively, there are two spaces of alternatives:

(42) $$H_{A}=\mathrm{span}\{|\;n\;\rangle \},\;\;H_{B}=\mathrm{span}\{|\;k\;\rangle \},$$

whose bases, in general, are different. Recollecting the existence of the subject space of mind $H_{S}$, we have the decision space

(43) $$H=H_{A}⨂H_{B}⨂H_{S}.$$

The decision-maker state is subject to the time evolution (9). The evolution operator satisfies the evolution equation (Equation (10)), now with the evolution generator

(44) $$\hat{H}\left(t\right)={\hat{H}}_{AS}\left(t\right)⨂{\hat{1}}_{B}\;+\;{\hat{1}}_{A}⨂{\hat{H}}_{BS}\left(t\right).$$

Again it is convenient to choose the basis composed of the eigenfunctions of the evolution generator, such that

(45) $$\hat{H}\left(t\right)\;|\;nk\alpha \;\rangle =E_{nk\alpha }\left(t\right)\;|\;nk\alpha \;\rangle .$$

This assumes the self-similarity of the decision maker in the form of the Lappo-Danilevsky condition (12), because of which we have

(46) $$\hat{U}\left(t\right)\;|\;nk\alpha \;\rangle =U_{nk\alpha }\left(t\right)\;|\;nk\alpha \;\rangle ,$$

where

(47) $$U_{nk\alpha }\left(t\right)=\exp\left\{-i\int_{0}^{t}E_{nk\alpha }\left(t^{\prime }\right)\;dt^{\prime }\right\}.$$

The joint probability that, first, a decision on $A_{n}$ is made (an event $A_{n}$ happens) and later a decision on $B_{k}$ is made (an event $B_{k}$ occurs) is defined as

(48) $$p\left(B_{k}A_{n},t\right)\equiv {\mathrm{Tr}}_{H}\;\hat{\rho }\left(t\right)\;\hat{P}\left(B_{k}\right)⨂\hat{P}\left(A_{n}\right).$$

This also can be written as

(49) $$p\left(B_{k}A_{n},t\right)\equiv {\mathrm{Tr}}_{AB}\;{\hat{\rho }}_{AB}\left(t\right)\;\hat{P}\left(B_{k}\right)⨂\hat{P}\left(A_{n}\right),$$

where

(50) $${\hat{\rho }}_{AB}\left(t\right)\equiv {\mathrm{Tr}}_{S}\;\hat{\rho }\left(t\right)=\sum_{\alpha }\langle \;\alpha \;|\;\hat{\rho }\left(t\right)\;|\;\alpha \;\rangle .$$

Expanding this results in the expression for the sought joint probability of choosing first the alternative $A_{n}$, and later, the alternative $B_{k}$, yields

(51) $$\begin{array}{c} p\left(B_{k}A_{n},t\right)=\sum_{n_{1}n_{2}}\;\sum_{k_{1}k_{2}}\;\sum_{\alpha }\;U_{n_{1}k_{1}\alpha }\left(t\right)\langle \;\alpha k_{1}n_{1}\;|\;\hat{\rho }\left(0\right)\;|\;n_{2}k_{2}\alpha \;\rangle \;U_{n_{2}k_{2}\alpha }^{*}\left(t\right)\times \\ \times \langle \;k_{2}\;|\;\hat{P}\left(B_{k}\right)\;|\;k_{1}\;\rangle \langle \;n_{2}\;|\;\hat{P}\left(A_{n}\right)\;|\;n_{1}\;\rangle . \end{array}$$

The process of making decisions is supposed to be smooth, such that its impact does not result in finite-time divergences,

(52) $$\left|\int_{t_{A}}^{t_{A}+\tau_{A}}E_{nk\alpha }\left(t\right)\;dt\;+\;\int_{t_{B}}^{t_{B}+\tau_{A}}E_{nk\alpha }\left(t\right)\;dt\;\right|\;<\;\infty .$$

The quantity $E_{nk\alpha }\left(t\right)$ characterizes the speed of the process of the subject deliberating about the given alternatives. For convenience, it is possible to define a rate parameter, entering the eigenvalue

(53) $$E_{nk\alpha }\left(t\right)=E_{nk\alpha }\left(t,g\right)$$

in such a way that a slow process would imply

(54) $$E_{nk\alpha }\left(t,g\right)\to 0\;\;\left(g\to 0\right),$$

while a fast process would mean that

(55) $$E_{nk\alpha }\left(t,g\right)\to \infty \;\;\left(g\to \infty \right).$$

When the process is slow, we have

(56) $$U_{nk\alpha }\left(t\right)≃1\;\;\left(g\to 0\right).$$

Then the corresponding probability becomes

(57) $$p\left(B_{k}A_{n},t\right)≃{\mathrm{Tr}}_{AB}\;{\hat{\rho }}_{AB}\left(0\right)\;\hat{P}\left(B_{k}\right)⨂\hat{P}\left(A_{n}\right)\;\;\left(g\to 0\right),$$

while the decision state reads as

(58) $${\hat{\rho }}_{AB}\left(t\right)≃{\hat{\rho }}_{AB}\left(0\right)\;\;\left(g\to 0\right).$$

It is useful to stress that even if at the initial moment of time the decisions on $A_{n}$ and $B_{k}$ formally look to be not explicitly connected, so that

$$\hat{\rho }\left(0\right)={\hat{\rho }}_{AS}\left(0\right)⨂{\hat{\rho }}_{BS}\left(0\right),$$

the decision state does not factorize anyway,

$${\hat{\rho }}_{AB}\left(0\right)={\mathrm{Tr}}_{S}\;{\hat{\rho }}_{AS}\left(0\right)⨂{\hat{\rho }}_{BS}\left(0\right),$$

being entangled through the subject’s mind. Only in the extreme, hardly realistic cases when at the initial moment of time all processes are absolutely not correlated, so that

$$\hat{\rho }\left(0\right)={\hat{\rho }}_{A}\left(0\right)⨂{\hat{\rho }}_{B}\left(0\right)⨂{\hat{\rho }}_{S}\left(0\right),$$

the joint probability factorizes:

$$p\left(B_{k}A_{n},0\right)=p\left(B_{k},0\right)\;p\left(A_{n},0\right).$$

Thus, even deciding on different alternatives, the total decision state, generally, remains entangled, thereby producing entangled states in the process of decision making [45]. The produced entanglement can be measured [46,47,48,49,50,51].

For a fast process, we have

(59) $$U_{n_{1}k_{1}\alpha }\left(t\right)\;U_{n_{2}k_{2}\alpha }\left(t\right)≃\delta_{n_{1}n_{2}}\delta_{k_{1}k_{2}}.$$

Then the decision state reduces to

(60) $${\hat{\rho }}_{AB}\left(t\right)≃\sum_{nk}{\hat{P}}_{n}⨂{\hat{P}}_{k}\;{\hat{\rho }}_{AB}\left(0\right)\;{\hat{P}}_{n}⨂{\hat{P}}_{k}\;\;\left(g\to \infty \right),$$

where

$${\hat{P}}_{n}=|\;n\;\rangle \langle \;n\;|,\;\;{\hat{P}}_{k}=|\;k\;\rangle \langle \;k\;|.$$

It is important to emphasize that the joint probability (48) is real-valued,

(61) $$p^{*}\left(B_{k}A_{n},t\right)=p\left(B_{k}A_{n},t\right).$$

It is not symmetric with respect to the interchange of $A_{n}$ and $B_{k}$, since the evolution generator (44) depends on the order of decisions, the first being $A_{n}$ and the second being $B_{k}$. As is postulated at the beginning of this section, at the time interval $\left[t_{A},t_{A}+\tau_{A}\right]$, a subject makes a decision on the alternatives $A_{n}$. Then, in the time interval $\left[t_{B}+\tau_{B}\right]$, where $t_{B}>t_{A}+\tau_{A}$, the subject decides on $B_{k}$. This means that the evolution generator can be presented as

$$\begin{array}{c} \hat{H}\left(t\right)=\left\{\begin{array}{cc} {\hat{H}}_{AS}\left(t\right)⨂{\hat{1}}_{B}, & t\leq t_{A}+\tau_{A}\\ {\hat{1}}_{A}⨂{\hat{H}}_{BS}\left(t\right), & t>t_{B}>t_{A}+\tau_{A} \end{array}\right.. \end{array}$$

From here its dependence on the order of decisions is evident. We may notice that the interchange of the decisions on $A_{n}$ and $B_{k}$ is similar to the inversion of time, hence

$$p\left(B_{k}A_{n},t\right)=p\left(A_{n}B_{k},-t\right).$$

Additionally, it is useful to compare the joint probability (49) with the Kirkwood distribution [52]. The latter is defined for two coinciding in time events, whose projectors pertain to the same space of alternatives $H_{A}=H_{B}$, which gives

$$p_{K}\left(B_{k}A_{n}\right)\equiv {\mathrm{Tr}}_{A}\;{\hat{\rho }}_{A}\;\hat{P}\left(B_{k}\right)\;\hat{P}\left(A_{n}\right).$$

It easy to see from the complex conjugate expression

$$p_{K}^{*}\left(B_{k}A_{n}\right)={\mathrm{Tr}}_{A}\;{\hat{\rho }}_{A}\;\hat{P}\left(A_{n}\right)\;\hat{P}\left(B_{k}\right)=p_{K}\left(A_{n}B_{k}\right)$$

that

$$p_{K}^{*}\left(B_{k}A_{n}\right)\neq p_{K}\left(B_{k}A_{n}\right),$$

which tells us that the Kirkwood distribution, generally, is complex-valued. Thus, the complex Kirkwood distribution and the real joint probability (49) are principally different.

## 6. Dual Decision Process

Subjects rarely make decisions based solely on the usefulness of alternatives, as prescribed by utility theory [9], especially when decisions are to be made under uncertainty. The choice between alternatives is practically always not purely objective and based on well defined values that could be quantified, but strong subjective feelings, biases, and heuristics are involved in the process of making decisions [53,54]. Intuition and emotions are also subjective, but they help us to make decisions, making them part of the decision-making process [55]. Taking account of subjective feelings and emotions in making decisions is important for the problem of human-computer interaction [56] and the creation of artificial intelligence [57,58].

Understanding that human decision making involves two sides, the rational evaluation of utility and intuitive irrational attraction or repulsion towards each of the alternatives has been thoroughly investigated and elucidated in the approach called dual-process theory [59,60,61,62]. Quantum techniques seem to be the most appropriate for portraying the duality of human decision processes, since quantum theory presupposes the so-called wave-particle duality. Below we show how the dual-process approach [59,60,61,62] is formulated in quantum language [10,46,63,64,65].

Thus, to describe decision making by real subjects it is necessary to take into account the *rational–irrational* duality of decision making. First, we need to say several words on the meaning of the notion “rational” that can be understood in different senses [66]. It is necessary to distinguish between the philosophical and psychological meanings of this term. In philosophical writings, one gives the definition of “rational” as all which leads to the desired goal [67]. However, under that definition, any illogical uncontrolled feeling that leads to the goal should be named rational. In decision making, one uses the *psychological* meaning of the term “rational” as what can be explained and evaluated logically and what follows explicitly formulated rules. On the contrary, emotions, intuition, and moral feelings cannot be logically and explicitly formulated and quantified, especially at the moment of making a decision. Thus, “rational” in decision making is what can be explicitly formulated, based on clear rules, deterministic, logical, prescriptive, normative. While “irrational” is just the opposite to “rational”.

In this way, one has to separate the psychological and philosophical definitions of “rational” or “irrational”. The psychological definition assumes that the distinction between “rational” and “irrational” is done at the moment of making a decision. Conversely, the philosophical definition of “rational” as what leads to the goal is a definition that can work better afterwards, when the goal has been reached. Only then it becomes clear what was leading to the goal and what was not. At the moment of making a decision, it is not always clear what leads to the goal and what does not.

The separation of decision making into rational and irrational has a simple and well defined psychological meaning based on actual physiological processes in the brain [68]. One often calls rational processes conscious, while irrational processes as subconscious.

From the mathematical point of view, the above can be formulated as follows. Let us consider the choice between a set of alternatives $A_{n}$. An alternative is represented by a vector $|A_{n}\rangle$ in a Hilbert space of alternatives $H_{A}$ and by a projector $\hat{P}\left(A_{n}\right)$ acting on this space. The subject space of mind is $H_{S}$. Thus, the decision space is

(62) $$H=H_{A}⨂H_{S}.$$

As is explained above, when choosing between alternatives, in addition to the rational evaluation of the utility of each alternative, the decision maker experiences irrational feelings. This implies that each alternative $A_{n}$, and its representation $|A_{n}\rangle$ in the space of alternatives $H_{A}$, is complimented by a set of subjective feelings $z_{n}$ represented by a vector $|z_{n}\rangle$ in the subject space of mind $H_{S}$. That is, in the process of decision making one compares not merely the alternatives $A_{n}$, but the composite prospects

(63) $$\pi_{n}\equiv A_{n}z_{n}.$$

Being a member of the subject space of mind, the vector $|z_{n}\rangle$ allows for the expansion

(64) $$|\;z_{n}\;\rangle =\sum_{\alpha }b_{n\alpha }\;|\;\alpha \;\rangle ,$$

in which $b_{n\alpha }$ are random quantities, which signifies a non-deterministic character of irrational feelings and emotions.

We do not impose an excessive number of conditions; instead we try to limit ourselves by the minimal number of restrictions. The required conditions will be imposed on the resulting expressions describing probability. Therefore, the vectors $|z_{n}\rangle$ are not forced to be obligatory normalized, so that the scalar product

$$\langle \;z_{n}\;|\;z_{n}\;\rangle =\sum_{\alpha }\;{\left|b_{n\alpha }\right|}^{2}$$

does not have to be one. Additionally, the vectors $|z_{n}\rangle$ with different labels are not orthogonal to each other. Respectively, the operator

(65) $$\hat{P}\left(z_{n}\right)\equiv |\;z_{n}\;\rangle \langle \;z_{n}\;|=\sum_{\alpha \beta }b_{n\alpha }b_{n\beta }^{*}\;|\;\alpha \;\rangle \langle \;\beta \;|$$

is not a projector, since

$$\hat{P}\left(z_{m}\right)\hat{P}\left(z_{n}\right)=\left(\;\sum_{\alpha }b_{m\alpha }^{*}b_{n\alpha }\right)\;|\;z_{m}\;\rangle \langle \;z_{n}\;|.$$

The prospect $A_{n}z_{n}$ is represented by the vector

(66) $$|\;A_{n}z_{n}\;\rangle =|\;A_{n}\;\rangle ⨂|\;z_{n}\;\rangle =\sum_{\alpha }b_{n\alpha }|\;A_{n}\alpha \;\rangle$$

in the decision space H. The prospect operator

(67) $$\hat{P}\left(A_{n}z_{n}\right)\equiv |\;A_{n}z_{n}\;\rangle \langle \;z_{n}A_{n}\;|=\hat{P}\left(A_{n}\right)⨂\hat{P}\left(z_{n}\right),$$

with the property

$$\hat{P}\left(A_{m}z_{m}\right)\hat{P}\left(A_{n}z_{n}\right)=\delta_{mn}\langle \;z_{n}\;|\;z_{n}\;\rangle \hat{P}\left(A_{n}z_{n}\right),$$

is not idempotent and is not a projector.

The resolution of unity

(68) $$\sum_{n}\hat{P}\left(A_{n}z_{n}\right)=1$$

is understood in the weak sense as the equality on average

(69) $$\langle \sum_{n}\hat{P}\left(A_{n}z_{n}\right)\rangle =1,$$

which implies

(70) $${\mathrm{Tr}}_{H}\;\hat{\rho }\left(t\right)\sum_{n}\hat{P}\left(A_{n}z_{n}\right)=1.$$

The extended version of the latter equality reads as

(71) $$\sum_{n}\;\sum_{\alpha \beta }b_{n\alpha }^{*}b_{n\beta }\;\langle \;\alpha A_{n}\;|\;\hat{\rho }\left(t\right)\;|\;A_{n}\beta \;\rangle =1.$$

The set of the operators $\left\{\hat{P}\left(A_{n}z_{n}\right)\right\}$ forms a kind of the positive operator-valued measure [69].

Thus, making a decision on an alternative $A_{n}$, a decision maker, as a matter of fact, considers a prospect $\pi_{n}=A_{n}z_{n}$, as far as, in addition to the rational quantification of the alternative utility, this alternative is subject to irrational subconscious evaluations. A decision maker not merely makes a decision on the usefulness of an alternative, but also experiences feelings of attraction or repulsion to this alternative. Thus, the probability of choosing an alternative is in fact a behavioral probability of the associated prospect comprising a decision on the utility and on the attractiveness of the alternative [65]. Keeping in mind that de facto, one practically always considers prospects associated with the given alternatives, and in order not to complicate notation, we shall denote the behavioral probability of a prospect

(72) $$p\left(\pi_{n},t\right)\equiv {\mathrm{Tr}}_{H}\;\hat{\rho }\left(t\right)\;\hat{P}\left(\pi_{n}\right)\equiv p\left(A_{n},t\right)$$

as identified with the alternative probability

(73) $$p\left(A_{n},t\right)={\mathrm{Tr}}_{H}\;\hat{\rho }\left(t\right)\;\hat{P}\left(A_{n}z_{n}\right).$$

The resolution of unity (70) or (71) guarantees the probability normalization

(74) $$\sum_{n}p\left(A_{n},t\right)=1,\;\;0\leq p\left(A_{n},t\right)\leq 1.$$

Separating in expression (73) diagonal terms

(75) $$f\left(A_{n},t\right)\equiv \sum_{\alpha }\;|\;b_{n\alpha }{\;|}^{2}\;\langle \;\alpha A_{n}\;|\;\hat{\rho }\left(t\right)\;|\;A_{n}\alpha \;\rangle$$

from off-diagonal terms

(76) $$q\left(A_{n},t\right)\equiv \sum_{\alpha \neq \beta }\;b_{n\alpha }^{*}b_{n\beta }\;\langle \;\alpha A_{n}\;|\;\hat{\rho }\left(t\right)\;|\;A_{n}\beta \;\rangle ,$$

we obtain the behavioral probability

(77) $$p\left(A_{n},t\right)=f\left(A_{n},t\right)+q\left(A_{n},t\right).$$

In the theory of quantum measurements, the diagonal term corresponds to classical theory, while the second, off-diagonal term is due to quantum interference and coherence. The transition from quantum to classical measurements and from quantum to classical probabilities is called decoherence [70]. Thus, the first term corresponds to a classical probability enjoying the properties

(78) $$\sum_{n}f\left(A_{n},t\right)=1,\;\;0\leq f\left(A_{n},t\right)\leq 1.$$

In decision theory, the classical probability is responsible for a rational choice of alternatives and can be named *rational fraction* or *utility fraction*, since its value is prescribed by the utility of the alternative [25,46,65,71,72].

The second term, entirely due to quantum effects, has the properties

(79) $$\sum_{n}q\left(A_{n},t\right)=0,\;\;-1\leq q\left(A_{n},t\right)\leq 1$$

following from Equations (77) and (78). The property (79) is named the alternation law [25,46,65,71,72]. From normalization (74), we also have

$$-f\left(A_{n},t\right)\leq q\left(A_{n},t\right)\leq 1-f\left(A_{n},t\right).$$

The quantum term *q* in decision theory is associated with irrational feelings characterizing the emotional attitude of the decision maker to the quality of considered alternatives, because of which it can be called *irrational factor*, *attraction factor*, or *quality factor* [25,46,65,71,72].

As is seen, for an alternative $A_{i}$ to be stochastically preferred over $A_{j}$, so that $p\left(A_{i}\right)>p\left(A_{j}\right)$, it is not always sufficient to be more useful, but it is necessary to be sufficiently attractive. An optimal alternative is that possessing the largest probability among the set of the considered alternatives.

For any generalization of decision theory, it is very important to include as a particular case the classical decision theory based on expected utility. Quantum decision theory [25,46,65,71,72] satisfies this requirement. The return to classical decision theory happens when the quantum term *q* tends to zero. This can be called the *quantum-classical correspondence principle*:

(80) $$p\left(A_{n},t\right)\to f\left(A_{n},t\right),\;\;q\left(A_{n},t\right)\to 0.$$

In the theory of quantum measurements, the disappearance of the quantum term, corresponding to quantum coherence, is called *decoherence*. The vanishing of this term can be due to the influence of surrounding environment [70,73,74,75] or to the action of measurements [35,76]. In decision theory, the decoherence can be caused by the influence of society providing information to the decision maker [77]. In both these cases, random disturbances from either surrounding or measurement devices, lead to the irreversibility of time arrow [78,79].

## 7. Evolution of Behavioral Probability

The temporal evolution of the behavioral probability (77) can be treated as in Section 3. The time dependence enters the matrix element

(81) $$\langle \;\alpha A_{n}\;|\;\hat{\rho }\left(t\right)\;|\;A_{n}\beta \;\rangle =\sum_{n_{1}n_{2}}U_{n_{1}\alpha }\left(t\right)\langle \;A_{n}\;|\;n_{1}\;\rangle \langle \;\alpha n_{1}\;|\;\hat{\rho }\left(0\right)\;|\;n_{2}\beta \;\rangle \;U_{n_{2}\beta }^{*}\left(t\right)\langle \;n_{2}\;|\;A_{n}\;\rangle .$$

If the process in the space of mind is asymptotically slow, then

$$U_{n\alpha }\left(t\right)≃1\;\;\left(g\to 0\right)$$

and we get

(82) $$\langle \;\alpha A_{n}\;|\;\hat{\rho }\left(t\right)\;|\;A_{n}\beta \;\rangle ≃\langle \;\alpha A_{n}\;|\;\hat{\rho }\left(0\right)\;|\;A_{n}\beta \;\rangle .$$

This means that

(83) $$f\left(A_{n},t\right)≃f\left(A_{n},0\right),\;\;q\left(A_{n},t\right)≃q\left(A_{n},0\right),$$

so that the probability practically does not change:

(84) $$p\left(A_{n},t\right)≃p\left(A_{n},0\right)\;\;\left(g\to 0\right).$$

In the opposite case of a fast process, when

$$U_{m\alpha }\left(t\right)\;U_{n\beta }^{*}\left(t\right)≃\delta_{mn}\delta_{\alpha \beta }\;\;\left(g\to \infty \right),$$

we obtain

(85) $$\langle \;\alpha A_{n}\;|\;\hat{\rho }\left(t\right)\;|\;A_{n}\beta \;\rangle ≃\delta_{\alpha \beta }\sum_{m}\;\langle \;A_{n}\;|\;m\;\rangle \langle \;\alpha m\;|\;\hat{\rho }\left(0\right)\;|\;m\alpha \;\rangle \langle \;m\;|\;A_{n}\;\rangle \;\;\left(g\to \infty \right).$$

Then the rational fraction reads as

(86) $$f\left(A_{n},t\right)≃\sum_{\alpha }\;|\;b_{n\alpha }{\;|}^{2}\;\langle \alpha A_{n}\;|\;\hat{\rho }\left(t\right)\;|\;A_{n}\alpha \;\rangle ,$$

where

(87) $$\hat{\rho }\left(t\right)=\sum_{m}{\hat{P}}_{m}\;\hat{\rho }\left(0\right)\;{\hat{P}}_{m}\;\;\left(g\to \infty \right),$$

while the quantum term, characterizing irrational feelings, vanishes:

(88) $$q\left(A_{n},t\right)≃0\;\;\left(g\to \infty \right).$$

Therefore, the behavioral probability reduces to the rational part,

(89) $$p\left(A_{n},t\right)≃f\left(A_{n},t\right)\;\;\left(g\to \infty \right).$$

Successive decision making can be described similarly to Section 5. For two successive decisions, one has to consider the probability

(90) $$p\left(B_{k}A_{n},t\right)={\mathrm{Tr}}_{H}\;\hat{\rho }\left(t\right)\;\hat{P}\left(B_{k}z_{k}\right)⨂\hat{P}\left(A_{n}z_{n}\right).$$

The above results demonstrate the essential dependence of the irrational term *q* from the speed of making decisions. The fact that, under a slow decision process, the probability practically does not vary, remaining close to that existing at the initial time, is not surprising. What is more interesting is that under a fast decision process, the quantum term vanishes. This can be explained as follows. The quantum term is caused by the interference and entanglement of subconscious feelings in the subject consciousness. In order that these processes happen, they need some time. However, in the case of extremely fast decision making, the subject just has no time for deliberation when the acts of coherence and entanglement could develop.

## 8. Evaluation of Initial Probability

Before considering any evolutional process, it is necessary to prescribe initial conditions corresponding to the initial moment of time that can be set as t=0. The initial probability

(91) $$p\left(A_{n}\right)\equiv p\left(A_{n},0\right)=f\left(A_{n}\right)+q\left(A_{n}\right)$$

is formed by the initial rational fraction and irrational factor,

(92) $$f\left(A_{n}\right)\equiv f\left(A_{n},0\right),\;\;q\left(A_{n}\right)\equiv q\left(A_{n},0\right).$$

To estimate the value of the initial rational fraction, it is possible to use the Luce rule [80], according to which, if the alternative $A_{n}$ is characterized by an attribute $a_{n}$, then the alternative weight can be written as

(93) $$f\left(A_{n}\right)=\frac{a_{n}}{\sum_{n}a_{n}}\;\;\left(a_{n}\geq 0\right).$$

For concreteness, let us study the case where alternatives are represented by lotteries

(94) $$A_{n}=\left\{x_{i},p_{n}\left(x_{i}\right):\;i=1,2,\ldots ,N_{n}\right\},$$

where $x_{i}$ are outcomes and $p_{n}\left(x_{i}\right)$ are the outcome probabilities normalized so that

$$\sum_{i}p_{n}\left(x_{i}\right)=1,\;\;0\leq p_{n}\left(x_{i}\right)\leq 1.$$

Employing a utility function u(x), one can define [9] the expected utility

(95) $$U\left(A_{n}\right)=\sum_{i}u\left(x_{i}\right)p_{n}\left(x_{i}\right).$$

The rational fraction describes the classical weight of an alternative that in the present case is connected with the expected utility in the following way [71,72]. If the expected utilities of all alternatives are semi-positive, they can be associated with the alternative attributes,

(96) $$a_{n}=U\left(A_{n}\right),\;\;U\left(A_{n}\right)\geq 0,$$

whereas if all utilities are negative, the alternative attributes can be defined by the inverse quantities

(97) $$a_{n}=\frac{1}{\left|\;U(A_{n})\;\right|},\;\;U\left(A_{n}\right)<0.$$

In the case wherein among the expected utilities there are both positive and negative utilities, it is possible to use the shift taking account of the available wealth $U_{0}$. This is done in the following way. Finding the minimal negative utility

$$U_{min}\equiv \min_{n}\;U\left(A_{n}\right)<0,$$

one defines

$$U_{0}\equiv \left|\;U_{min}\;\right|$$

interpreted as the wealth available to decision makers before they make decisions. Then the shifted utilities

(98) $$\bar{U}\left(A_{n}\right)\equiv U\left(A_{n}\right)+U_{0}\geq 0$$

are used instead of $U(A_{n})$, and we return to the case of semi-positive expected utilities.

The so defined rational fraction, or utility fraction, enjoys the natural properties: For semi-positive utilities, the fraction tends to zero, when its utility tends to zero,

(99) $$f\left(A_{n}\right)\to 0,\;\;U\left(A_{n}\right)\to +0,$$

and tends to one, when its utility is very large,

(100) $$f\left(A_{n}\right)\to 1,\;\;U\left(A_{n}\right)\to +\infty .$$

For negative expected utilities, we have

(101) $$f\left(A_{n}\right)\to 1,\;\;U\left(A_{n}\right)\to -0,$$

and respectively,

(102) $$f\left(A_{n}\right)\to 0,\;\;U\left(A_{n}\right)\to -\infty .$$

When considering empirical data, the rational fraction characterizes the fraction of decision makers making decisions on the basis of rational normative rules. A more elaborate expression for the rational fraction can be derived by employing the minimization of an information functional [65,71,72].

The irrational factor *q*, as has been explained above, is a random quantity distributed over the interval [−1,1]. However, being a random variable does not preclude it to possess a typical average quantity. The ways of defining a non-informative prior for *q* are described in [46,65,71,72]. Below we present a slightly modified derivation of the non-informative prior for the irrational factor.

Let the related distribution function be denoted as φ(x). This distribution is normalized

(103) $$\int_{-1}^{1}\varphi \left(x\right)\;dx=1.$$

Then the average value of a positive irrational factor is defined as

(104) $$q_{+}=\int_{0}^{1}x\varphi \left(x\right)\;dx.$$

Respectively, the average value of a negative factor is

(105) $$q_{-}=\int_{-1}^{0}x\varphi \left(x\right)\;dx.$$

**Proposition** **1.** *The non-informative priors for the average irrational factors are*

(106) $$q_{+}=\frac{1}{4},\;\;q_{-}=-\frac{1}{4}.$$

**Proof.**  *W*ith the notation

(107) $$\lambda_{+}=\int_{0}^{1}\varphi \left(x\right)\;dx,\;\;\lambda_{-}=\int_{-1}^{0}\varphi \left(x\right)\;dx,$$

the normalization condition (103) takes the form

(108) $$\lambda_{+}+\lambda_{-}=1,\;\;0\leq \lambda_{\pm }\leq 1.$$

In the average irrational factors (104) and (105), *x* is a monotonic function, while φ(x) is integrable. Therefore, employing the theorem of average yields

(109) $$q_{+}=x_{+}\lambda_{+},\;\;q_{-}=x_{-}\lambda_{-},$$

where

(110) $$0\leq x_{+}\leq 1,\;\;-1\leq x_{-}\leq 0.$$

As a non-informative prior for the values of $x_{\pm }$ and $\lambda_{\pm }$ one takes the averages over the domains of their definition, that is,

(111) $$x_{+}=\frac{1}{2},\;\;x_{-}=-\frac{1}{2},\;\;\lambda_{\pm }=\frac{1}{2}.$$

Substituting this into quantities (104) and (105) proves equalities (106). □

This property is termed the *quarter law*. The mentioned above alternation law and the quarter law can be used for estimating the aggregate values of the behavioral probabilities according to the rule

(112) $$p\left(A_{n}\right)=f\left(A_{n}\right)\pm 0.25.$$

The sign of the irrational factor is prescribed in accordance to the alternative being attractive or repulsive [46,65,71,72].

This approach was employed for explaining a number of paradoxes in classical decision making [46,63,64,71,72,81,82] and was found to be in good agreement with a variety of experimental observations [65,71,72,83].

## 9. Quantum Intelligence Network

In the previous sections, we have considered quantum decision making by subjects that act independently of each other. Even then the probability of alternatives could vary with time due to the entangling properties of the evolution operator inducing entanglement in the decision space [50,51,84], in addition to that caused by the initial subject state of mind [85]. The situation becomes much more involved when subjects exchange their information with each other. When there is a society of subjects interacting through the exchange of information, we obtain a network. Since the agents make decisions, we have an intelligence network. The probabilities of alternatives will vary owing to the informational interaction. From empirical investigations, it is known that decision makers do alter their decisions as a result of mutual charing of information [86,87,88,89,90,91,92,93]. This type of intelligence networks plays an important role as prolegomena into the problem of artificial intelligence [94,95,96].

Assume that we are considering a society of *N* agents enumerated by the index i=1,2,…,N, who decide with respect to alternatives $A_{n}$, with $n=1,2,\ldots ,N_{A}$. Thus, the probability for an *i*-th agent deciding on an alternative $A_{n}$ at the moment of time *t* is $p_{i}\left(A_{n},t\right)$. Each probability satisfies the standard normalization

(113) $$\sum_{n=1}^{N_{A}}\;p_{i}\left(A_{n},t\right)=1,\;\;0\leq p_{i}\left(A_{n},t\right)\leq 1.$$

Accordingly, the related rational fraction $f_{i}\left(A_{n},t\right)$ is normalized as

(114) $$\sum_{n=1}^{N_{A}}\;f_{i}\left(A_{n},t\right)=1,\;\;0\leq f_{i}\left(A_{n},t\right)\leq 1.$$

And for the irrational factor, one has

(115) $$\sum_{n=1}^{N_{A}}\;q_{i}\left(A_{n},t\right)=0,\;\;-f_{i}\left(A_{n},t\right)\leq q_{i}\left(A_{n},t\right)\leq 1-f_{i}\left(A_{n},t\right).$$

To give a realistic description for the temporal evolution of an intelligence network, it is required to take into account that decision making needs some time after receiving information. Denoting this delay time as τ, allows us to represent the dynamics of a behavioral probability as

(116) $$p_{i}\left(A_{n},t+\tau \right)=f_{i}\left(A_{n},t\right)+q_{i}\left(A_{n},t\right).$$

The rational fraction weakly varies with time, so that on the time scale shorter than the discounting time [97] it can be treated as constant. Its value is prescribed by the utilities of the given alternatives, as is explained in Section 8.

The time dependence of the irrational factor can be derived following Section 7, similarly to the derivation of the coherent interference term in the theory of quantum measurements, where the role of irrational subconscious feelings is played by the random influence of environment [35,76,78,79,98]. If a decision maker at time *t* accumulates the amount of information $M_{i}\left(t\right)$ from other members of the society, then the irrational factor is discounted from its initial value, becoming equal to

(117) $$q_{i}\left(A_{n},t\right)=q_{i}\left(A_{n},0\right)\exp\left\{-M_{i}\left(t\right)\right\}.$$

The amount of the accumulated information composes [77,99] the information-memory functional

(118) $$M_{i}\left(t\right)=\sum_{t^{\prime }=1}^{t}\;\sum_{j=1}^{N}\;J_{ij}\left(t,t^{\prime }\right)\mu_{ij}\left(t^{\prime }\right),$$

in which $J_{ij}\left(t,t^{\prime }\right)$ is the interaction-memory function and $\mu_{ij}\left(t\right)$ is the information gain by a subject *i* from a subject *j* at time *t*. To exclude self-action, one has to set either $J_{ii}=0$ or $\mu_{ii}=0$. At the initial moment of time there is no yet additional information, so that one has the initial condition

(119) $$M_{i}\left(0\right)=0.$$

The information gain can be modeled by the Kullback-Leibler [100,101] relative information

(120) $$\mu_{ij}\left(t\right)=\sum_{n=1}^{N_{A}}\;p_{i}\left(A_{n},t\right)\;\ln\;\frac{p_{i}\left(A_{n},t\right)}{p_{j}\left(A_{n},t\right)}.$$

The information gain (120) is semi-positive, $\mu_{ij}\geq 0$, due to the inequality lnx≥1−1/x. As is evident, $\mu_{ii}=0$. Depending on the range of the interactions between the agents and the longevity of their memory, there can arise different situations whose typical examples are as follows.

*Long-range interactions* have the form

(121) $$J_{ij}\left(t,t^{\prime }\right)=\frac{1}{N-1}\;J\left(t,t^{\prime }\right)\;\;\left(i\neq j\right).$$

*Short-range interactions* act only between the nearest neighbors,

(122) $$J_{ij}\left(t,t^{\prime }\right)=J\left(t,t^{\prime }\right)\delta_{\langle ij\rangle },$$

where $\delta_{<ij>}$ equals one for *i* and *j* being the nearest neighbours and is zero otherwise.

*Long term memory*, that lasts forever, does not depend on time,

(123) $$J_{ij}\left(t,t^{\prime }\right)=J_{ij}.$$

*Short-term memory*, on the contrary, corresponds to the situation, when only the last step is remembered,

(124) $$J_{ij}\left(t,t^{\prime }\right)=J_{ij}\delta_{tt^{\prime }}.$$

Combining these ultimate cases gives us the following four possibilities for the information-memory functional.

**Long-range interactions and long-term memory**:

(125) $$M_{i}\left(t\right)=\frac{J}{N-1}\sum_{t^{\prime }=1}^{t}\;\sum_{j=1}^{N}\;\mu_{ij}\left(t^{\prime }\right).$$

**Long-range interactions and short-term memory**:

(126) $$M_{i}\left(t\right)=\frac{J}{N-1}\sum_{j=1}^{N}\;\mu_{ij}\left(t\right).$$

**Short-range interactions and long-term memory**:

(127) $$M_{i}\left(t\right)=J\sum_{t^{\prime }=1}^{t}\;\sum_{j=1}^{N}\;\delta_{\langle ij\rangle }\mu_{ij}\left(t^{\prime }\right).$$

**Short-range interactions and short-term memory**:

(128) $$M_{i}\left(t\right)=J\sum_{j=1}^{N}\;\delta_{\langle ij\rangle }\mu_{ij}\left(t\right).$$

Keeping in mind modern human societies, we have to accept that long-range interactions are more realistic. This is because the modern-day information exchange practically does not depend on the location of interacting agents who are able to exchange information through phone, Skype, Zoom, etc.

## 10. Case of Two Alternatives

A very frequent situation is when agents have to decide between two suggested alternatives, that is, when $N_{A}=2$. Then it is straightforward to simplify the notation by setting

(129) $$\begin{array}{c} p_{i}\left(A_{1},t\right)\equiv p_{i}\left(t\right),\;\;p_{i}\left(A_{2},t\right)=1-p_{i}\left(t\right),\\ f_{i}\left(A_{1},t\right)\equiv f_{i}\left(t\right),\;\;f_{i}\left(A_{2},t\right)=1-f_{i}\left(t\right),\\ q_{i}\left(A_{1},t\right)\equiv q_{i}\left(t\right),\;\;q_{i}\left(A_{2},t\right)=-q_{i}\left(t\right). \end{array}$$

Now the dynamics is governed by the equation

(130) $$p_{i}\left(t+\tau \right)=f_{i}\left(t\right)+q_{i}\left(t\right),$$

with the irrational factor

(131) $$q_{i}\left(t\right)=q_{i}\left(0\right)\exp\left\{-M_{i}\left(t\right)\right\}.$$

The information gain takes the form

(132) $$\mu_{ij}\left(t\right)=p_{i}\left(t\right)\;\ln\;\frac{p_{i}\left(t\right)}{p_{j}\left(t\right)}+\left[1-p_{i}\left(t\right)\right]\;\ln\;\frac{1-p_{i}\left(t\right)}{1-p_{j}\left(t\right)}.$$

As is seen, $\mu_{ii}=0$. Setting initial conditions, it is necessary to obey the restriction

$$-f_{i}\left(0\right)\leq q_{i}\left(0\right)\leq 1-f_{i}\left(0\right).$$

Another realistic simplification could be when the considered society consists of two types of agents essentially differing by their initial decisions. Then the question is: How the initial decisions would vary with time being caused by the mutual exchange of information? Will agents with different initial decisions come to a consensus, as it often happens after a number of interactions [102,103]?

When the society can be divided into two parts of typical agents, with each part having similar initial decisions within the group, but essentially different initial decisions between the groups, the situation becomes equivalent to the consideration of two typical agents with these different initial decisions. As is explained above, long-range interactions are more realistic for the information exchange in the modern society. Below we present the results of numerical calculations for this type of interactions. The behavior of the society strongly depends on the type of memory the agents have.

**Long-term memory**. In the case of long-term memory, the information-memory functionals for two groups are

(133) $$M_{1}\left(t\right)=J\sum_{t^{\prime }=1}^{t}\;\mu_{12}\left(t^{\prime }\right),\;\;M_{2}\left(t\right)=J\sum_{t^{\prime }=1}^{t}\;\mu_{21}\left(t^{\prime }\right).$$

The rational fractions are kept constant in time and the parameters are set J=1 and τ=1. The society dynamics is strongly influenced by the initial decisions. There can happen two types of behavior depending on the relations between the initial rational fractions and irrational factors.

(i) *Rational group conventions*. There is the rational-irrational accordance in the initial choice of both groups. Then at the initial moment of time, one group, say the first group, estimates the utility of the first alternative higher than the second group. Taking account of irrational feelings keeps the same preference with respect to behavioral probabilities:

(134) $$f_{1}\left(0\right)>f_{2}\left(0\right),\;\;p_{1}\left(0\right)>p_{2}\left(0\right).$$

Recall that $f_{i}\left(t\right)\equiv f_{i}\left(A_{1},t\right)$ and $p_{i}\left(t\right)\equiv p_{i}\left(A_{1},t\right)$. Respectively, if the first group estimates the utility of the first alternative lower than the second group, the irrational feelings do not change this preference:

(135) $$f_{1}\left(0\right)<f_{2}\left(0\right),\;\;p_{1}\left(0\right)<p_{2}\left(0\right).$$

In the case of this rational-irrational accordance, independently of initial conditions, the behavioral probabilities tend to the respective rational fractions:

(136) $$p_{i}\left(t\right)\to f_{i}\left(0\right)\;\;\left(t\to \infty \right).$$

(ii) *Common convention*. At the initial time, the inequalities between the rational fractions of the groups and the inequalities between their behavioral probabilities are opposite with each other, that is, one has either

(137) $$f_{1}\left(0\right)>f_{2}\left(0\right),\;\;p_{1}\left(0\right)<p_{2}\left(0\right),$$

or

(138) $$f_{1}\left(0\right)<f_{2}\left(0\right),\;\;p_{1}\left(0\right)>p_{2}\left(0\right).$$

In this case, the behavioral probabilities tend with time to the common convention:

(139) $$p_{i}\left(t\right)\to p^{*}\;\;\left(t\to \infty \right)$$

approximately equal to

(140) $$p^{*}=\frac{f_{1}\left(0\right)q_{2}\left(0\right)-f_{2}\left(0\right)q_{1}\left(0\right)}{q_{2}\left(0\right)-q_{1}\left(0\right)}.$$

These results can be interpreted in the following way. In the situation of the rational-irrational accordance, the decision makers are more rational, while irrational feelings, such as emotions, play less important role. Under the prevalence of rational arguments, the agents are inclined to choose the alternative with a higher utility.

In the case of the rational-irrational discordance, the decision makers are forced to more efficiently exchange information, as a result of which they manage to develop a mutual convention.

**Short-term memory**. In this case, the information-memory functionals are

(141) $$M_{1}\left(t\right)=J\mu_{12}\left(t\right),\;\;M_{2}=J\mu_{21}\left(t\right).$$

We set again J=1 and τ=1. Numerical solution of the evolution equations reveals the existence of two types of possible dynamics.

(i) *Group conventions*. The behavioral probabilities for each group tend with time to their own limits not coinciding with the corresponding rational fractions:

(142) $$p_{i}\left(t\right)\to p_{i}^{*}\;\;\left(t\to \infty \right).$$

(ii) *Everlasting fluctuations*. The behavioral probabilities for both groups do not tend to any fixed point, but demonstrate everlasting oscillations. The details of the above numerical solutions can be found in [99].

In a society with short-term memory, there is no enough accumulated information for the formation of a common convention. Each group in such a society either develops their own goal, not necessarily rational, or constantly fluctuates without elaborating a consensus.

## 11. Dynamic Decision Inconsistencies

There are several so-called dynamic or time inconsistencies in decision making characterizing situations in which a decision-maker’s preferences change over time in such a way that a preference at one moment of time can become inconsistent with a preference at another point in time. Below we consider some of these inconsistencies and show that they find quite natural explanations in QDT.

### 11.1. Question Order Bias

The order that several questions are asked in a survey or study can influence the answers that are given as much as by 40% [104,105]. That is because the human brain has a tendency to organize information into patterns. The earlier questions may provide information that subjects use as context in formulating their subsequent answers, or affect their thoughts, feelings, and attitudes towards the questioned problem. Sociological research gives a number of illustrations of this bias [105]. Thus, from a December 2008 poll we know that when people were asked, “All in all, are you satisfied or dissatisfied with the way things are going in this country today?” immediately after having been asked, “Do you approve or disapprove of the way George W. Bush is handling his job as president?” 88 percent said they were dissatisfied, compared with only 78 percent without the context of the prior question.

In classical probability theory, the probability of joint events is symmetric. This is contrary to the quantum decision theory. Assume an interrogator first asks a question *A*, so that the answer suggests two alternatives: yes ($A_{1}$) or no ($A_{2}$). After this, the interrogator poses another question *B*, also with the possible answers: yes ($B_{1}$) or no ($B_{2}$). As follows from Section 5, the probability $p(A_{i}B_{j})$ does not equal the probability $p(B_{j}A_{i})$, since they are defined through different decision states. The situation is similar to that occurring in the problem of quantum contextuality [106,107,108], where two random variables ($A_{i}B_{j}$ and $B_{j}A_{i})$ cannot be characterized by a single density matrix.

In that way, taking account of dynamic evolution in QDT shows that, in general, the alternatives are not commutative, in the sense that $p\left(A_{i}B_{j}\right)\neq p\left(B_{j}A_{i}\right)$, which is in agreement with the empirical data.

### 11.2. Planning Paradox

In classical decision theory, there is the principle of dynamic consistency according to which a decision taken at one moment of time should be invariant in time, provided no new information has become available and all other conditions are not changed. Then a decision maker, preferring an alternative at time $t_{1}$ should retain the choice at a later time $t_{2}>t_{1}$. However, this principle is often broken, which is called the effect of dynamic inconsistency.

A stylized example of dynamic inconsistency is the planning paradox, when a subject makes a plan for the future, while behaving contrary to the plan as soon as time comes to accomplish the latter. A typical case of this inconsistency is the stop-smoking paradox [109,110]. A smoker, well understanding the damage to health caused from smoking, plans to stop smoking in the near future, but time goes by, “the future” comes; however, the person does not stop smoking. Numerous observations [109] show that 85% of smokers do plan to stop smoking in the near future; however, only 36% really stop smoking during the next year after making the plan. It is possible to pose the question: would it be feasible to predict the percentage of subjects who will really stop smoking during the next year, knowing that at the present time 85% of them plan on doing this. Below we show that QDT allows us to make this prediction [63].

Let us denote by $A_{1}$ the alternative to stop smoking in the near future, and by $A_{2}$, the alternative to not stop smoking. Let us denote by $B_{1}$ the decision to really stop smoking, and by $B_{2}$, the decision of refusing to really stop smoking. According to QDT, the corresponding probabilities are

$$p\left(A_{1}\right)=f\left(A_{1}\right)+q\left(A_{1}\right),\;\;p\left(A_{2}\right)=f\left(A_{2}\right)+q\left(A_{2}\right),$$

$$p\left(B_{1}\right)=f\left(B_{1}\right)+q\left(B_{1}\right),\;\;p\left(B_{2}\right)=f\left(B_{2}\right)+q\left(B_{2}\right).$$

The utility factors do not change in time, so that the utility of planning to stop in the near future is the same as the utility to stop in reality and the utility to not stop in the near future is the same as that of not stopping in reality:

$$f\left(A_{1}\right)=f\left(B_{1}\right),\;\;f\left(A_{2}\right)=f\left(B_{2}\right).$$

Planning to stop smoking, subjects understand the attractiveness of this due to health benefits. Hence the average attraction factors, according to Section 8, are

$$q\left(A_{1}\right)=\frac{1}{4},\;\;q\left(A_{2}\right)=-\frac{1}{4}.$$

But as soon as one has to stop smoking in reality, one feels uneasy from the necessity to forgo the pleasure of smoking, because of which the attraction factors become

$$q\left(B_{1}\right)=-\frac{1}{4},\;\;q\left(B_{2}\right)=\frac{1}{4}.$$

This is to be complemented by the normalization conditions

$$p\left(A_{1}\right)+p\left(A_{2}\right)=1,\;\;p\left(B_{1}\right)+p\left(B_{2}\right)=1,$$

$$f\left(A_{1}\right)+f\left(A_{2}\right)=1,\;\;f\left(B_{1}\right)+f\left(B_{2}\right)=1.$$

Since 85% of subjects plan to stop smoking, we have

$$p\left(A_{1}\right)=0.85,\;\;p\left(A_{2}\right)=0.15.$$

Solving the above equations, the fraction of subjects who will really stop smoking is predicted to be

$$p\left(B_{1}\right)=0.35,\;\;p\left(B_{2}\right)=0.65.$$

This is in beautiful agreement with the observed fraction of smokers really stopping smoking during the next year after making the decision [109],

$$p_{exp}\left(B_{1}\right)=0.36,\;\;p_{exp}\left(B_{2}\right)=0.64.$$

Thus, knowing only the percentage of subjects planning to stop smoking, it is straightforward, by means of QDT, to predict the fraction of those who will stop smoking in reality. This case is also of interest because it gives an example of preference reversal: when planning to stop smoking, the relation between the probabilities is reversed as compared to the relation between the fractions of those who have really stopped smoking,

$$p\left(A_{1}\right)>p\left(A_{2}\right),\;\;p\left(B_{1}\right)<p\left(B_{2}\right).$$

### 11.3. Disjunction Effect

The disjunction effect is the violation of the sure-thing principle [111]. This principle states: *if the alternative $A_{1}$ is preferred to the alternative $A_{2}$, when an event $B_{1}$ occurs, and it is also preferred to $A_{2}$, when an event $B_{2}$ occurs, then $A_{1}$ should be preferred to $A_{2}$, when it is not known which of the events, either $B_{1}$ or $B_{2}$, has occurred*. This principle is easily illustrated for classical probability. Let $B=B_{1}+B_{2}$ be the alternative when it is not known which of the events, either $B_{1}$ or $B_{2}$, has occurred. For a classical probability, one has

$$f\left(A_{n}B\right)=f\left(A_{n}B_{1}\right)+f\left(A_{n}B_{2}\right)\;\;\left(n=1,2\right).$$

From here, it immediately follows that if

$$f\left(A_{1}B_{1}\right)>f\left(A_{2}B_{1}\right),\;\;f\left(A_{1}B_{2}\right)>f\left(A_{2}B_{2}\right),$$

then

$$f\left(A_{1}B\right)>f\left(A_{2}B\right).$$

However, empirical studies have discovered numerous violations of the sure-thing principle, which was called the disjunction effect [112]. Such violations are typical for two-step composite games of the following structure. First, a group of agents takes part in a game, where each agent can either win (event $B_{1}$) or lose (event $B_{2}$), with equal probability 0.5. They are then invited to participate in a second game, having the right either to accept the second game (event $A_{1}$) or to refuse it (event $A_{2}$). The second stage is realized in different variants: One can either accept or decline the second game under the condition of knowing the result of the first game. Or one can either accept or decline the second game without knowing the result of the first game. The probabilities, as usual, are understood in the frequentist sense as the fractions of individuals making the corresponding decisions [99].

From the experiment of Tversky and Shafir [112], we have

$$f\left(A_{1}B_{1}\right)=0.345,\;\;f\left(A_{1}B_{2}\right)=0.295,$$

$$f\left(A_{2}B_{1}\right)=0.155,\;\;f\left(A_{2}B_{2}\right)=0.205.$$

This shows that the alternative $A_{1}B$ is more useful than $A_{2}B$, since

$$f\left(A_{1}B\right)=0.64,\;\;f\left(A_{2}B\right)=0.36,$$

which seems to agree with the sure-thing principle. However, $f(A_{n}B)$ is not yet the whole probability that reads as

$$p\left(A_{n}B\right)=f\left(A_{n}B\right)+q\left(A_{n}B\right)\;\;\left(n=1,2\right).$$

Making a decision to play, without knowing the result of the first game, is less attractive, because of which $q(A_{1}B)=-1/4$, while $q(A_{2}B)=1/4$. As a result, we find

$$p\left(A_{1}B\right)=0.39,\;\;p\left(A_{2}B\right)=0.61,$$

which is in good agreement with the empirical data of Tversky and Shafir [112],

$$p_{exp}\left(A_{1}B\right)=0.36,\;\;p_{exp}\left(A_{2}B\right)=0.64.$$

The dynamic consistency of the disjunction effect can be analyzed as in Section 9 and Section 10. Details can be found in [99], where it is shown that, when decision makers are allowed to exchange information, the absolute values of the attraction factors diminish with time. This conclusion also is in good agreement with empirical observations [113].

## 12. Dynamic Preference Intransitivity

Transitivity is of central importance to both psychology and economics. It is the cornerstone of normative and descriptive decision theories [9]. Individuals, however, are not perfectly consistent in their choices. When faced with repeated choices between alternatives *A* and *B*, people often choose in some instances *A* and *B* in others. Such inconsistencies are observed even in the absence of systematic changes in the decision maker’s taste, which might be due to learning or sequential effects. The observed inconsistencies of this type reflect inherent variability or momentary fluctuation in the evaluative process. Then preference should be defined in a probabilistic fashion [80]. Nevertheless, there occur several choice situations where time transitivity may be violated even in a probabilistic form. Then one says that there is dynamic preference intransitivity [114,115,116,117,118,119,120].

The occurrence of preference intransitivity depends on the considered decision model and on the accepted definition of transitivity. But the general meaning is as follows. Suppose one evaluates three alternatives A, B, C, considering them in turn by pairs. One compares *A* and *B*, and, according to the selected definition of preference, concludes that *A* is preferred over *B*, which can be denoted as A>B. Then one compares *B* and *C*, finding that B>C. Finally, comparing *C* and *A*, one discovers that C>A. This results in the preference loop A>B>C>A signifying the intransitivity effect.

As a simple illustration of the intransitivity effect, we can adduce the Fishburn [116] example. Imagine that a person is about to change jobs. When selecting a job, the person evaluates the suggested salary and the prestige of the position. There are three choices: job *A*, with the salary 65,000$, but low prestige; job *B*, with the salary 58,000$ and medium prestige; and job *C*, with the salary 50,000$ and high prestige. The person chooses *A* over *B* because of the better salary, and with a small difference in prestige, *B* over *C* because of the same reason; and comparing *C* and *A*, the person prefers *C* because of the higher prestige, although a lower salary. Thus, one comes to the preference loop A>B>C>A.

Let us show how this problem can be resolved in QDT. Recall that the definition of the behavioral probability (72) is contextual, in the sense that it depends on the initial conditions for the decision state and on the given time. This means that the comparison of each pair of alternatives constitutes a separate contextual choice, even if the external conditions remain unchanged. The utility factors can be calculated as described in Section 8. Thus, considering the pair *A* and *B* in the Fishburn example, we have the utility factors

$$f_{1}\left(A\right)=0.528,\;\;f_{1}\left(B\right)=0.472,$$

where the label marks the moment of time $t_{1}$ and an initial condition ${\hat{\rho }}_{1}\left(t_{1}\right)$ for the decision state. Due to the close prestige of the both jobs, their attraction factors coincide, which, as follows from the alternation law in Section 6, gives

$$q_{1}\left(A\right)=q_{1}\left(B\right)=0.$$

This leads to the behavioral probabilities

$$p_{1}\left(A\right)=0.528,\;\;p_{1}\left(B\right)=0.472.$$

Since $p_{1}\left(A\right)>p_{1}\left(B\right)$, the job *A* at time $t_{1}$ is stochastically preferred over *B*.

Similarly, comparing the jobs *B* and *C* at time $t_{2}$, we get the utility factors

$$f_{2}\left(B\right)=0.537,\;\;f_{2}\left(C\right)=0.463,$$

and the attraction factors

$$q_{2}\left(B\right)=q_{2}\left(C\right)=0.$$

Hence, the probabilities are

$$p_{2}\left(B\right)=0.537,\;\;p_{2}\left(C\right)=0.463,$$

which implies that *B* at time $t_{2}$ is stochastically preferred over *C*.

In the comparison of the jobs *C* and *A* at time $t_{3}$, we find the utility factors

$$f_{3}\left(C\right)=0.435,\;\;f_{3}\left(A\right)=0.565.$$

Now the positions are of a very different quality, so that the attraction factors are

$$q_{3}\left(C\right)=\frac{1}{4},\;\;q_{3}\left(A\right)=-\frac{1}{4}.$$

Therefore, the probabilities become

$$p_{3}\left(C\right)=0.685,\;\;p_{3}\left(A\right)=0.315.$$

Then, at time $t_{3}$, the job *C* is stochastically preferred over *A*.

However, we should not forget that these comparisons were accomplished at different moments of time and under different initial conditions. Therefore, there is nothing extraordinary such that differently defined probabilities can be intransitive. Even more, there are arguments [121] that such intransitivities can be advantageous for alive beings in the presence of irreducible noise during neural information processing.

The arising preference loops can be broken in two ways. First, the process of comparison requires time during which there can appear additional information. The attraction factors, as is explained in Section 9 and Section 10, vary with time, diminishing as time increases. When $q_{3}\left(C\right)$ and $q_{3}\left(A\right)$ tend to zero then

$$p_{3}\left(C\right)\;⟶\;f_{3}\left(C\right)=0.435,$$

$$p_{3}\left(A\right)\;⟶\;f_{3}\left(A\right)=0.565.$$

Since now $p_{3}\left(C\right)<p_{3}\left(A\right)$, *A* becomes preferred over *C*; hence, the preference loop is broken.

The other very natural way is as follows. As soon as there appears a preference loop, this implies that decisions at different moments of time and under different contexts should not be compared. One has to reconsider the whole problem at one given moment of time *t*. Considering all three alternatives A,B,C in the frame of one given choice, we have

f(A)=0.376,  f(B)=0.335,  f(C)=0.289.

The classification of the related qualities can be estimated according to the QDT rule

$$q\left(A\right)=-\frac{1}{4},\;\;q\left(B\right)=0,\;\;q\left(C\right)=\frac{1}{4}.$$

Then we find

p(A)=0.126,  p(B)=0.335,  p(C)=0.539,

which establishes the relation (A≺B≺C) between all alternatives, and no problems or paradoxes arise.

## 13. Conclusions

The basic ideas of quantum decision theory are presented, with the emphasis on the problems of time evolution of decision processes. The relations between operationally testable events and behavioral features of making decisions are elucidated. The interplay of rational and irrational sides of decision making is explained. The approach to describing quantum intelligence networks is developed. As illustrations, several time inconsistencies are analyzed.

The main points of quantum decision theory are based on the techniques used in the theory of quantum measurements. Therefore, the presented approach can be employed for characterizing evolutional processes in quantum measurements. The behavior of many self-organizing complex systems is similar to decision making [122], because of which the approach could be useful in applications to complex systems and to the problems of artificial intelligence.
